# P-42. Clinical Evaluation of Combination Cefazolin and Ertapenem for Persistent Methicillin-Susceptible Staphylococcus aureus Bloodstream Infections

**DOI:** 10.1093/ofid/ofaf695.271

**Published:** 2026-01-11

**Authors:** Kimberly Le, Meagan Adamsick O'Brien, Alyssa P Gould, Bria Benson, Robert Crawford

**Affiliations:** Novant Health Presbyterian Medical Center, Charlotte, North Carolina; Novant Health Presbyterian Medical Center, Charlotte, North Carolina; Novant Health, Charlotte, North Carolina; Novant Health Forsyth Medical Center, Winston Salem, North Carolina; Novant Health Forsyth Medical Center, Winston Salem, North Carolina

## Abstract

**Background:**

*Staphylococcus aureus* continues to be a main cause of bloodstream infections (BSI) with an annual incidence rate of fifty cases per 100,000 person-years and mortality rates up to 30%. Methicillin-susceptible strains are frequently considered less complicated, however, pose just as high mortality rates and therapeutic challenges as methicillin-resistant cases. First-line treatment continues to be anti-staphylococcal penicillins, like nafcillin or oxacillin, and first generation cephalosporins, such as cefazolin. In cases of persistent bacteremia, newer *in vitro* studies and small case series have shown possible synergistic activity when cefazolin and ertapenem are combined. The purpose of this study is to describe the use of combination cefazolin and ertapenem in the treatment of persistent BSIs due to methicillin-susceptible *Staphylococcus aureus* (MSSA).

**Table 1.**
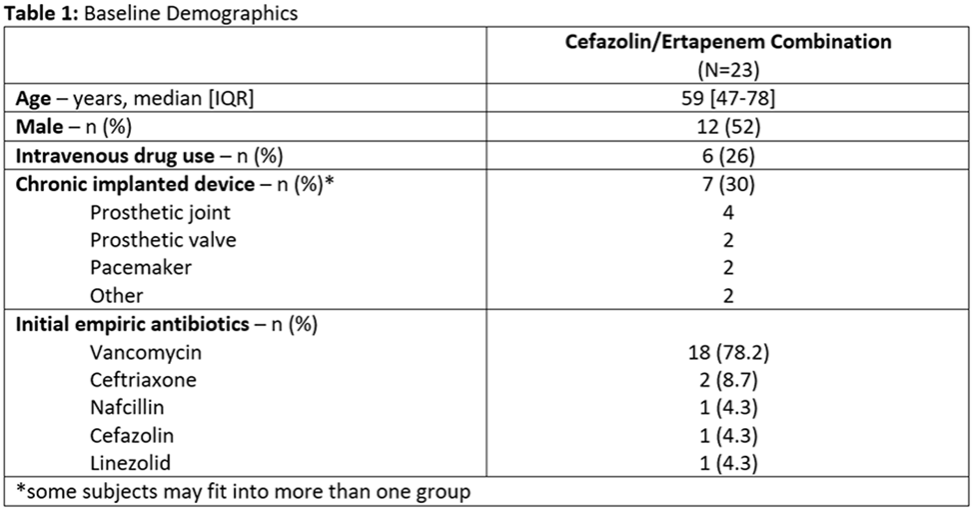
Baseline Demographics

**Table 2.**
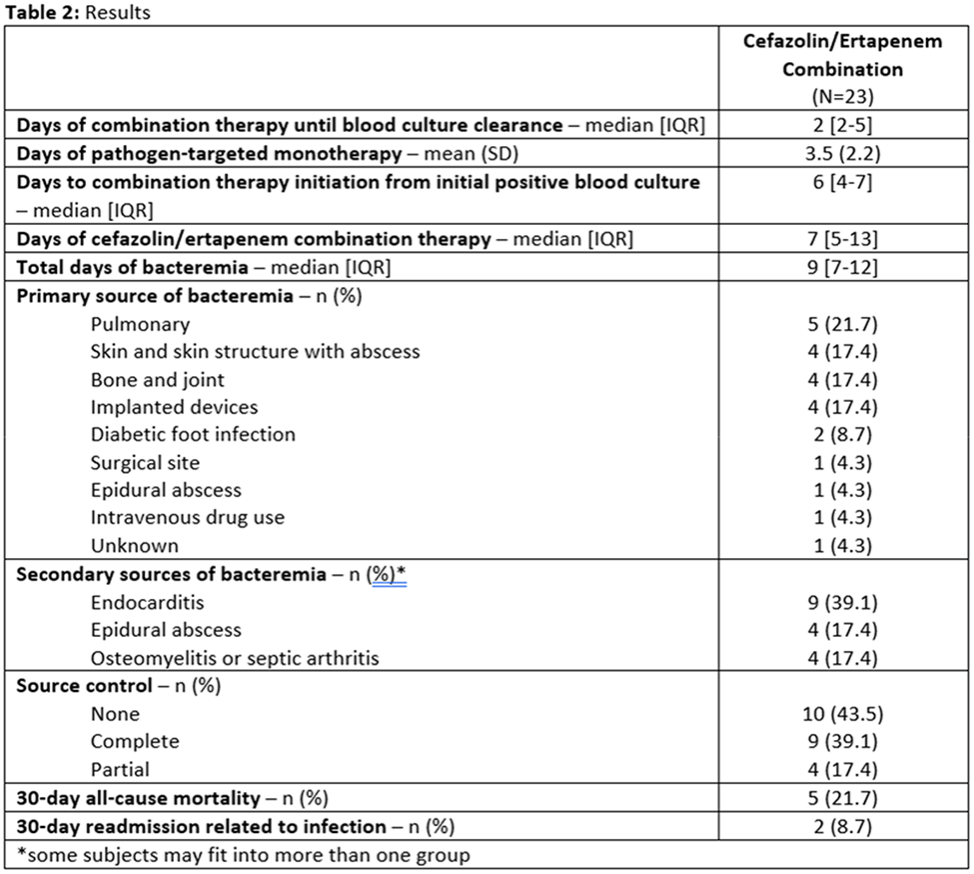
Results

**Methods:**

This case series was conducted through an electronic medical record report identifying patients ≥18 years old with MSSA BSI who received concomitantly at least one dose of both cefazolin and ertapenem at Novant Health acute care facilities from January 1, 2021, through July 31, 2024.

**Results:**

Twenty-three patients were included. The most common sources of BSI included pulmonary, skin and skin structure with abscess, bone and joint, and implanted device-related infections. The median blood culture clearance was seen after 2 days of combination cefazolin and ertapenem. The average days of monotherapy was 3.5 days and the median days from the initial positive blood culture to combination therapy initiation was 6 days. 30-day all-cause mortality occurred in 5 patients (21.7%), which was consistent with mortality rates previously reported for persistent Staphylococcus aureus BSI.

**Conclusion:**

In the data-lacking clinical challenge of persistent MSSA BSI, combination cefazolin and ertapenem may serve as a safe and effective way to improve the time to clearance of blood cultures. This is the largest study to date summarizing the clinical success of this novel combination strategy. Additional data is needed to compare this regimen to standard of therapy; however, clinicians can consider utilizing this combination strategy in patients with persistent MSSA BSI.

**Disclosures:**

All Authors: No reported disclosures

